# 23. An adverse neuropsychiatric reaction following treatment with hydroxychloroquine: a case report

**DOI:** 10.1093/rap/rky033.014

**Published:** 2018-09-20

**Authors:** Saadia S Ali, Hugh Jones

**Affiliations:** Rheumatology, Kingston Hospital, London, UNITED KINGDOM

**Introduction:** Hydroxychloroquine is a synthetic antimalarial drug derived from 4-aminoquinolone. Its use is widespread in rheumatology, particularly in the treatment of mild to moderately active rheumatoid arthritis and non-life threatening manifestations of systemic lupus erythematosus (SLE). Antimalarials are among the safest drugs used in the treatment of rheumatic diseases. The most common adverse reactions involve the skin, gastrointestinal tract and central nervous system. Adverse effects on the CNS include headaches, dizziness and increased anxiety. Rare complications include psychosis and convulsions. We present a case of an 82 year old Caucasian female who presented to older age psychiatry services with new onset of confusion, unusual behaviour and change of personality shortly after commencement of hydroxychloroquine, for treatment of rheumatoid arthritis. She had complete resolution of symptoms following cessation of this medication.

**Case description:** An 82 year old Caucasian female diagnosed with seropositive, CCP positive rheumatoid arthritis in 2009 presented to rheumatology with a persistent pruritus, which she felt was secondary to her methotrexate. As her DAS 28 score was 3.04, it was decided in conjunction with the patient to stop methotrexate and commence hydroxychloroquine at 200 mg once daily (dosed at 4 mg/kg/day). It should be noted that she had normal renal and liver function on routine blood testing. Two weeks after commencement of hydroxychloroquine, the patient started demonstrating out of character behaviours including phoning the ambulance to repair her blood pressure machine and being physically aggressive and verbally abusive to her neighbours. This included an incident where she was escorted out of a shopping centre as she was physically aggressive to other customers. In addition she stopped eating and as a result lost 14 kg in weight over three months. She was investigated by older age psychiatric services for a possible underlying psychosis or an emerging frontotemporal dementia. She was first investigated with a CT head scan, which showed no acute intracranial abnormality. She had no evidence of cognitive impairment on Addenbrooke’s Cognitive Examination. MRI head showed some mild generalised volume loss with proportional volume loss in the hippocampal heads but there was no evidence of neoplasm or cerebrovascular disease. Psychometric testing on a range of cognitive assessments showed that the patient’s scores fell within the expected ranges with no evidence of cognitive difficulties. Confusion blood testing including calcium, syphilis screening, thyroid function, B12 and folate levels were all unremarkable. As a result of her erratic behaviour the DVLA was informed and her driver’s licence revoked on medical grounds.

Following withdrawal of hydroxychloroquine, which took place three months after the onset of symptoms, the patient’s behaviour and aggressive tendencies returned to normal within three weeks. This patient's past medical history is remarkable for chronic obstructive lung disease, diverticulosis and bilateral cataracts. She is currently on tiotropium bromide, symbicort and Ad Cal D3. She was not started on any new medications apart from hydroxychloroquine in the last six months. She has since re-started her methotrexate at 12.5 mg along with folic acid for her rheumatoid arthritis.

**Discussion:** Hydroxychloroquine is metabolised in the liver by dealkylation to desethyl chloroquine and bisedesethyl chloroquine. It has an elimination half-life of approximately 40 days owing to reduced clearance due to its large volume of distribution in tissues. Some authors have suggested that in the brain, hydroxychloroquine can have a tissue concentration that is 10 times higher than plasma concentration. The exact mechanism by which hydroxychloroquine causes neuropsychiatric side effects remains unknown. It is postulated that premorbid personality is a likely predisposing factor. One author stipulates that lysosomal dysfunction caused by hydroxychloroquine may be a possible mechanism in causing psychiatric symptoms. Other hypothesised mechanisms include the cholinergic imbalance with acetylcholine reduction and even the down regulation of P-glycoprotein at the blood brain barrier brought on by hydroxychloroquine. This case is unique as it represents only the second reported case in the literature of neuropsychiatric manifestations of hydroxychloroquine in a patient with rheumatoid arthritis. Overall there are only four reported cases of psychosis due to hydroxychloroquine in the literature.

Given the contemporaneous onset of symptoms following initiation with hydroxychloroquine and the return to her normal mental state after withdrawal of the drug, it is likely that her psychiatric manifestations were the result of hydroxychloroquine initiation. In addition, formal extensive cognitive testing, neuropsychological assessments, brain scans and blood testing failed to show an alternative cause for the patient’s symptoms.

**Key Learning Points:** While it is important that alternative causes for psychosis be investigated, hydroxychloroquine should always be considered as a causative agent in the differential diagnosis of a patient presenting with new onset neuropsychiatric symptoms. Even in patients dosed at normal therapeutic ranges (4 mg/kg/day), neuropsychiatric manifestations may still occur. From the literature review, premorbid personality may be a predisposing factor in the development of psychosis following hydroxychloroquine initiation and this information should be sought before this medication is prescribed.

**Disclosure: S.S. Ali:** None. **H. Jones:** None.

